# Synthesis vs. salvage of ester- and ether-linked phosphatidylethanolamine in the intracellular protozoan pathogen *Toxoplasma gondii*

**DOI:** 10.1038/s42003-023-04664-x

**Published:** 2023-03-22

**Authors:** Bingjian Ren, Xiaohan Liang, Jos F. Brouwers, Rosalba Cruz Miron, Bang Shen, Nishith Gupta

**Affiliations:** 1 Department of Molecular Parasitology, Faculty of Life Sciences, Humboldt University, Berlin, Germany; 2 State Key Laboratory of Agricultural Microbiology, Huazhong Agricultural University, Wuhan, China; 3 Research Group for Analysis Techniques in the Life Sciences, School of Life Sciences and Technology, Avans University of Applied Sciences, Breda, The Netherlands; 4 Intracellular Parasite Education and Research Labs (iPEARL), Department of Biological Sciences, Birla Institute of Technology and Science, Pilani (BITS-P), Hyderabad, India

**Keywords:** Parasite biology, Lipidomics

## Abstract

*Toxoplasma* gondii is a prevalent zoonotic pathogen infecting livestock as well as humans. The exceptional ability of this parasite to reproduce in several types of nucleated host cells necessitates a coordinated usage of endogenous and host-derived nutritional resources for membrane biogenesis. Phosphatidylethanolamine is the second most common glycerophospholipid in *T. gondii*, but how its requirement in the acutely-infectious fast-dividing tachyzoite stage is satisfied remains enigmatic. This work reveals that the parasite deploys de novo synthesis and salvage pathways to meet its demand for ester- and ether-linked PtdEtn. Auxin-mediated depletion of the phosphoethanolamine cytidylyltransferase (ECT) caused a lethal phenotype in tachyzoites due to impaired invasion and cell division, disclosing a vital role of the CDP-ethanolamine pathway during the lytic cycle. In accord, the inner membrane complex appeared disrupted concurrent with a decline in its length, parasite width and major phospholipids. Integrated lipidomics and isotope analyses of the *Tg*ECT mutant unveiled the endogenous synthesis of ester-PtdEtn, and salvage of ether-linked lipids from host cells. In brief, this study demonstrates how *T. gondii* operates various means to produce distinct forms of PtdEtn while featuring the therapeutic relevance of its de novo synthesis.

## Abstract (web summary)

A genetic and lipidomic investigation reveals that depletion of ECT in *Toxoplasma gondii* disrupts ester-phosphatidylethanolamine (PtdEtn) synthesis and results in parasite death. Notably, *T. gondii* salvages ether-PtdEtn from its host cells.

## Introduction

The protozoan phylum Apicomplexa comprises many common intracellular pathogens, such as *Toxoplasma*, *Plasmodium*, *Eimeria* and *Cryptosporidium*. *Toxoplasma gondii*—the only known species in the genus—has a remarkable ability to infect many types of nucleated cells in various vertebrates; it is therefore recognized as one of the most successful pathogens. The natural infection of the hosts by *T. gondii* starts by ingesting the oocysts or cysts in the contaminated food. The sporozoite and bradyzoite stages released from the oocyst or cyst, respectively, infect the gastric epithelium and further develop into a highly infectious, promiscuous and fast-dividing tachyzoite stage, which reproduces in other tissues, eventually causing necrosis by perpetual lytic cycles^1,2^. Upon physicochemical and immune stress, some tachyzoites differentiate into encysted bradyzoites, establishing chronic infection. For intracellular reproduction within host cells, the parasite must produce sufficient membrane biomass *via* its synthesis and salvage pathways, many of which bestow excellent anti-parasitic therapeutic targets^3,4^.

Glycerophospholipids constitute a major ingredient of biomembranes in tachyzoites of *T. gondii*^4–8^. Phosphatidylcholine (PtdCho), phosphatidylethanolamine (PtdEtn), phosphatidylthreonine (PtdThr), phosphatidylserine (PtdSer), phosphatidylinositol (PtdIns), phosphatidylglycerol (PtdGro) and phosphatidate (PtdOH) are typical phospholipids present in tachyzoites and required for the optimal lytic cycle^5,7,9–12^. Besides their customary roles for parasite replication, many phospholipids have recently emerged as key players in calcium homeostasis and signal transduction, contributing to the gliding motility, invasion and egress of tachyzoites^7,12–15^. The parasite is able to synthesize phospholipids using precursors acquired from the host cell^5,7,9–12,16–19^. Further, it can salvage certain phospholipid species/probes from the extracellular and/or intracellular milieu^12,18,20^.

PtdEtn is the second most common phospholipid in tachyzoites, present in ester and ether-linked forms^5–7^. Several routes can produce ester-PtdEtn, including distinct PtdSer decarboxylases (PSDs) located in the parasite mitochondrion and parasitophorous vacuole, CDP-ethanolamine *a.k.a*. Kennedy pathway in the endoplasmic reticulum, and *via* P4-ATPase-mediated flipping in the plasma membrane^5,10,20,21^. Its endogenous synthesis through the CDP-ethanolamine pathway requires a diacylglycerol scaffold that can be generated by the tachyzoites^18^. Although not yet studied in *T. gondii*, ether-linked PtdEtn contains alkyl-glycerol backbone made by an alkyl-glycerone phosphate synthase in mammalian cells^22^. In functional terms, the conical shape of ester-PtdEtn contributes to the membrane curvature, regulating the budding, fusion and fission events and thereby facilitating the membrane-protein interactions^23^. On the other hand, the shape of ether-PtdEtn allows stronger intermolecular hydrogen bonding between headgroups, resulting in decreased membrane fluidity^22,24^.

This work examined the biogenesis and physiological relevance of ester- and ether-PtdEtn during the lytic cycle of *Toxoplasma* tachyzoites. We studied the phosphoethanolamine cytidylyltransferase (ECT), which catalyzes ethanolamine kinase-derived phosphoethanolamine to CDP-ethanolamine in the parasite cytosol. Our data show that ECT is essential for the asexual reproduction of tachyzoites in human cells. Its conditional depletion by mutagenesis impairs the amount and synthesis of ester-PtdEtn species, whereas ether-PtdEtn remains unaffected. Likewise, we discovered that intracellular parasites could salvage ether-linked PtdEtn from the host cells.

## Results

### *T. gondii* encodes a putative ethanolamine cytidylyltransferases

**Fig. 1:**
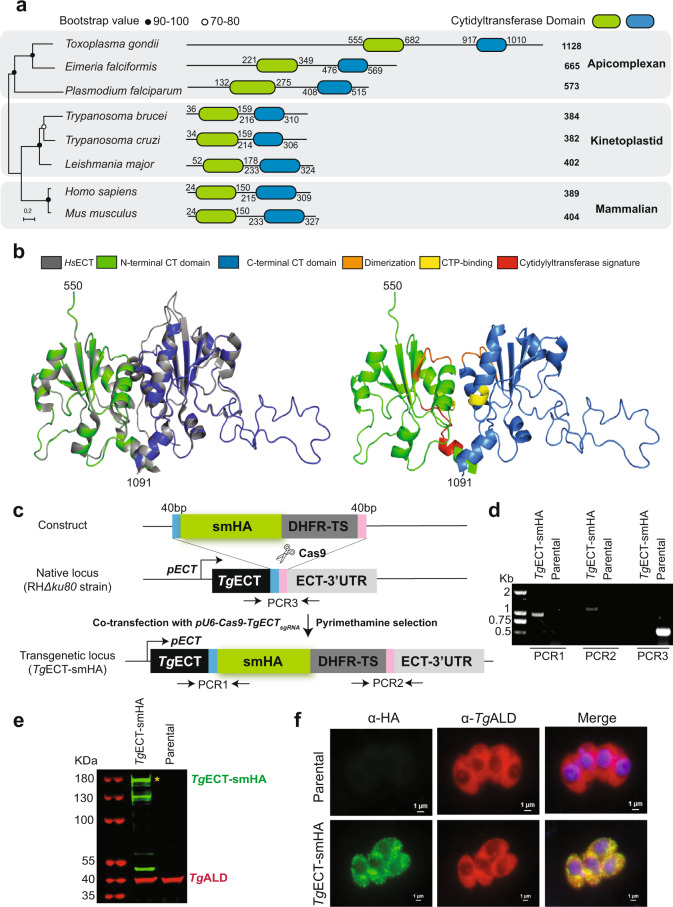
Toxoplasma encodes an ethanolamine cytidylyltransferase located in the cytosol. **a** Primary structure and phylogenetic proximity of ethanolamine cytidylyltransferases from representative parasitic protists and mammalian hosts. The protein sequences retrieved from the Uniprot or NCBI databases were aligned by Clustal W, and the phylogram was constructed by the MEGA 11 software (maximum likelihood method, JTT matrix model). The alignment of ECTs can be seen in Supplemental Fig. 1. **b** A three-dimensional homology model of the cytidylyltransferase domains in *Tg*ECT (*right*) and its superimposition with the crystal structure of *Hs*ECT (gray, PDB: 3ELB, *left*). **c** Scheme indicating the 3′-genomic tagging of *Tg*ECT with a smHA epitope. **d** Genomic PCR confirming the integration of smHA tag and DHFR-TS selection marker at the ECT locus. PCR1 and PCR2 were used to test the transgenic locus, while PCR3 was included as a control for the parental strain. **e** Immunoblot showing the expression of smHA-tagged *Tg*ECT in the transgenic strain. The asterisk (*) depicts the correct *Tg*ECT-smHA band (180-kDa). Other lower-size bands are possible degradation products of *Tg*ECT-smHA. **f** Immunofluorescent co-localization of *Tg*ECT-smHA with *Tg*ALD in tachyzoites (24 h post-infection). Parasitized cultures were labeled using α-HA and α-*Tg*ALD antibodies.

Our former work has reported the occurrence of a functional CDP-ethanolamine pathway in the tachyzoites^5^, albeit we could not identify a potential alkyl-glycerone phosphate synthase in the parasite genome. In the following studies, we described the first and last enzymes of de novo PtdEtn synthesis, i.e., ethanolamine kinase (EK) and ethanolamine phosphotransferase^9,10^. Here, we identified the ECT protein, which uses phosphoethanolamine and CTP as the substrates to produce CDP-ethanolamine^25^. By comparing with homologs from the model organisms, *Saccharomyces cerevisiae*, *Homo sapiens*, *Mus musculus* and *Trypanosoma brucei*, we found a potential ECT in the *Toxoplasma* genome (TGGT1_310280, Fig. 1). Unlike mammalian and kinetoplastid ECTs, which encompass about 400 residues and form separate clades, apicomplexan homologs are much longer, forming their own distinct clade. *Pf*ECT and *Ef*ECT encode 573 and 665 residues, respectively, while *Tg*ECT contains 1128 amino acids. Apicomplexan ECTs possess an unusual N-terminal extension of >100 residues compared to canonical homologs. *Tg*ECT is exceptional with a long extension of about 500 amino acids, resulting in a 3× larger protein than its non-apicomplexan counterparts (Fig. 1a).

*Tg*ECT harbors two tandem cytidylyltransferase domains of *circa* 100–150 residues parted by a linker region, analogous to its counterparts in other organisms. This contrasts with the bacterial CTP: glycerol-3-phosphate cytidylyltransferase or eukaryotic CTP: phosphocholine cytidylyltransferase (CCT), which contains only a single CT domain. Sequence alignment of the typical ECT domains also identified conserved residues (Supplemental Fig. 1). All signature motifs of the cytidylyltransferase family are present in *Tg*ECT: HSGH and HVGH; an RTEGISTS motif; KWVDEVI and RVVDEVI regions (Supplemental Fig. 1). Homology modeling of *Tg*ECT domains based on the *Hs*ECT structure (PDB code, 3ELB) suggests that the HSGH, HVGH and RTEGISTS are involved in forming a catalytic center, while the KWVDEVI and RVVDEVI enable the dimerization of the N- and C-terminal CT domains (Fig. 1b). The N-terminal CT domain in *Hs*ECT and *Pf*ECT are critical for their catalytic activity^26,27^, which is likely to hold true for *Tg*ECT (Fig. 1a).

### *Tg*ECT is a cytosolic protein refractory to genetic deletion in tachyzoites

The first step of the CDP-ethanolamine pathway producing phosphoethanolamine occurs in the cytosol^9^, and the third/last reaction synthesizing PtdEtn happens in the endoplasmic reticulum^10^. Here, we examined the localization of ECT to decipher the subcellular location of CDP-ethanolamine formation. We generated a transgenic parasite strain expressing ECT fused to a C-terminal spaghetti monster (10xHA) by 3′-genomic tagging. A donor amplicon with the 5′ and 3′ homology sequences flanking the pyrimethamine-resistant DHFR-TS expression cassette (selection marker) was co-transfected into tachyzoites with a CRISPR construct encoding Cas9 and a gene-specific *sg*RNA (Fig. 1c). The drug-resistant parasites were cloned by limiting dilution and screened by genomic PCR for integration at the desired locus (Fig. 1d). In addition, we confirmed the successful tagging by western blot, revealing an expected band of 180 kDa corresponding to *Tg*ECT-smHA in transgenic but not in the parental strain (Fig. 1e). Immunofluorescent staining revealed a punctate distribution of ECT-smHA within the parasite, co-localizing with a known cytosolic marker, aldolase (*Tg*Ald)^28,29^ (Fig. 1f). To assess the physiological relevance of ECT during the lytic cycle, we attempted to delete the gene in tachyzoites *via* CRISPR/Cas9-assisted double homologous recombination. However, our multiple experiments to achieve a viable knockout mutant were futile, implying that this protein is indispensable for tachyzoite survival.

### Conditional knockdown of ECT reveals its essentiality in tachyzoites

**Fig. 2:**
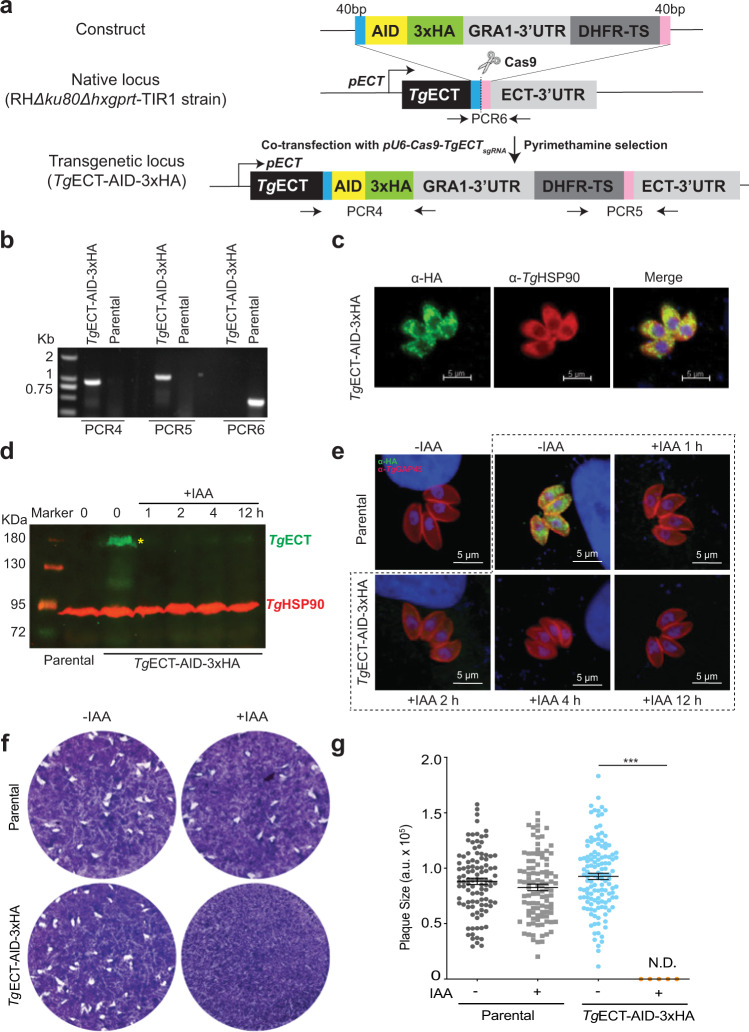
Conditional depletion of *Tg*ECT ablates the lytic cycle of T. gondii tachyzoites. **a** The CRISPR/Cas9-mediated 3′-tagging of the *Tg*ECT gene with an auxin-inducible degron and 3xHA. The *pU6-Cas9-TgECT*_*sgRNA*_ construct (encoding for Cas9 and ECT-specific *sg*RNA) was transfected with a donor amplicon (AID-3xHA-3′UTR_Gra1_-DHFR-TS flanked by 40 bp 5′/3′-homology arms) into the RHΔ*ku80*-Δ*hxgprt*-TIR1 parental strain. Pyrimethamine-resistant tachyzoites expressing DHFR-TS were cloned and screened by PCR. The eventual *Tg*ECT-AID-3xHA strain allowed conditional downregulation of ECT by Indole-3-acetic acid (IAA). **b** Recombination**-**specific screening PCR to decipher the integration of AID-3xHA at the ECT locus (see primers in **a**). PCR4/PCR5 and PCR6 indicate the testing of transgenic and native loci, respectively. **c** Immunofluorescent images confirming the co-localization of *Tg*ECT and *Tg*HSP90 in the mutant. **d**, **e** IAA-dependent expression of AID-3xHA-tagged ECT shown by immunoblot and immunofluorescence methods. Parasites were cultured in the absence or presence of 100 μM IAA for the indicated time periods. Immunostaining was done using α-HA and α-*Tg*HSP90 (**d**) or α-HA and α-*Tg*GAP45 (**e**) antibodies. Each sample’s equivalent amount of protein was resolved on 8% SDS-PAGE for blotting, followed by immunostaining. **f**, **g** Plaques produced by *Tg*ECT-AID-3xHA and parental strains (−/+IAA). Crystal violet-stained images reveal plaques (irregular white area) formed by successive lytic cycles of the stated strains in confluent HFF monolayers (blue). The plaque size, as quantified by ImageJ, is presented in arbitrary units (*a. u*.). 150–200 plaques of each strain were scored (*n* = 3 assays; means ± S.E.; ***p ≤ 0.001).

In the following work, we made a conditional mutant of *Tg*ECT based on an auxin-induced degron, which directs the tagged proteins to proteasomal degradation upon incubation with indole-3-acetic acid (IAA)^30^. A CRISPR/Cas9 construct encoding Cas9 and *sg*RNA to cleave the ECT-3′UTR was co-transfected with an amplicon for homology-directed repair at the target locus in tachyzoites. The donor sequence contained 5′ and 3′ homology arms (40 bp each) flanking the AID-3xHA motif for 3′-insertional tagging and DHFR-TS selection marker (Fig. 2a). The genomic integration was examined by PCR screening using crossover-specific primers (Fig. 2a, b), confirming the 3′-gene tagging, which was verified by DNA sequencing. Consistent with *Tg*ECT-smHA (Fig. 1f), AID-3xHA tagged ECT was present in the cytosol colocalizing with *Tg*HSP90, another cytoplasmic marker^31^ (Fig. 2c). Immunoblot revealed a protein of ∼180 kDa corresponding to AID-3xHA-tagged ECT in the transgenic strain cultured without IAA, while the signal can be rapidly depleted within 1 h of IAA treatment (Fig. 2d). The fast depletion of ECT by IAA was confirmed by immunofluorescence assay (Fig. 2e, Supplemental Fig. 2).

A conditional mutant enabled us to evaluate the importance of ECT for the parasite’s lytic cycle in HFF cells. We set up plaque assays, which indicate the overall growth fitness of *T. gondii* (Fig. 2f, g). The parental strain exhibited normal growth regardless of IAA in culture, and so did the *Tg*ECT-AID-3xHA mutant in the absence of auxin. In contrast, the IAA-induced depletion of ECT arrested the parasite growth, as evident by the lack of plaques in the host-cell monolayer. These results resonate with the preceding observation that the ECT gene cannot be deleted, ascertaining its physiologically-critical role in *T. gondii*.

### *Tg*ECT is required for the parasite invasion and proliferation

**Fig. 3:**
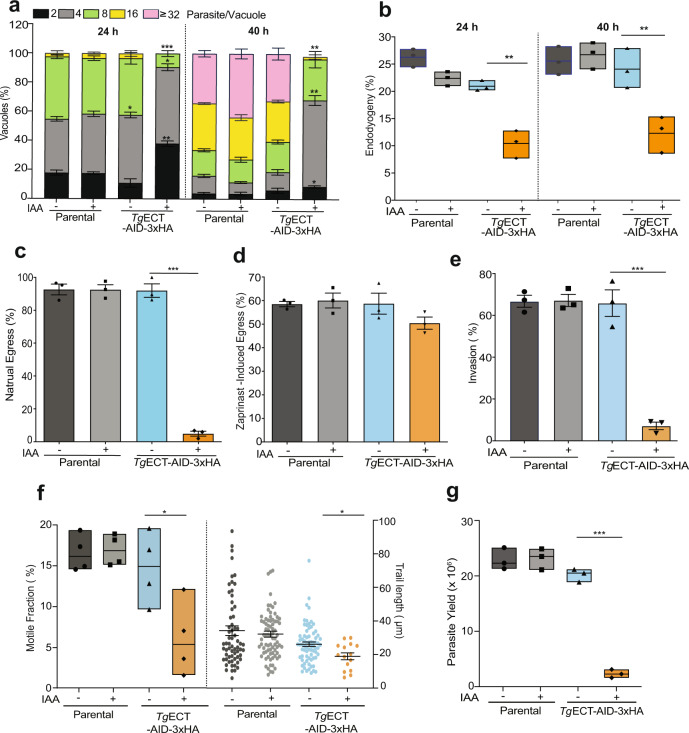
*Tg*ECT is needed for the motility-dependent invasion and the cell division of tachyzoites. **a**, **b** The replication and the budding efficiency of the *Tg*ECT-AID-3xHA and parental strains (−/+IAA). *Tg*GAP45-stained tachyzoites replicating within vacuoles were plotted (parasites/vacuole) to reflect the proliferation rate of each strain (**a**). For a simplified bar graph with data points, refer to Supplemental Fig. 6. The budding of daughter cells was calculated based on the quantification of *Tg*IMC3-positive vacuoles harboring tachyzoites with progeny (**b**). The bar graphs are based on 400–500 vacuoles for each sample. **c**, **d** Egress rates of the ECT mutant and parental strains in −/+ IAA conditions. For natural (**c**) and zaprinast-induced (**d**) egress, results were obtained by counting 30 random fields with >200 parasitophorous vacuoles. **e** Invasion efficiency of the indicated strains (−/+IAA). A total of >1000 events were scored for the shown bar graphs. **f** Gliding motility of tachyzoites. *Tg*SAG1-stained parasites were analyzed for the motile fraction (200–400 cells, *n* = 4 assays) and trail length (>60 trails per group, except for IAA-treated *Tg*ECT-AID-3xHA (13 trails)). **g** The yield of tachyzoites (−/+IAA). HFF monolayers (10^6^ cells) were infected with indicated strains (MoI = 1) and cultured for 48 h with or without IAA, followed by parasite counting. The (**a**–**g**) show data from *n* = 3 or more experiments (means ± S.E.; *p ≤ 0.05, **p ≤ 0.01, ***p ≤ 0.001).

We next examined the cause of a lethal phenotype in ECT-depleted tachyzoite cultures by additional assays, such as the replication, endodyogeny (cell budding), egress, invasion and gliding motility (Fig. 3). For the first two experiments, we chose time points corresponding to early (24 h) and late (40 h) parasitized cultures (Fig. 3a, b). The replication was quantified by enumerating ‘parasites per vacuole’ and calculating the fraction of vacuoles with variable numbers of progeny. Unlike the parental strain, which remained unaffected by IAA, the ECT mutant had a much higher fraction of small vacuoles (1–8 parasites) in auxin-treated cultures at both time points compared to untreated samples (Fig. 3a). The large vacuoles with >16 parasites were barely present under ECT-depleted conditions, contrasting with the control cultures. In agreement, the assessment of endodyogeny demonstrated a strongly impaired daughter cell formation only in the IAA-treated *Tg*ECT-AID-3xHA mutant (Fig. 3b).

The conditional mutant also exhibited a defect in natural egress following the completion of tachyzoite replication (Fig. 3c). We scored induced egress in response to zaprinast to test whether the observed phenotype was due to slow replication and not due to impaired egress signaling. This potent phosphodiesterase inhibitor triggers a premature exit of intracellular tachyzoites by activating the cGMP signaling^32,33^. Indeed, the egress phenotype was rescued by zaprinast (Fig. 3d), implying an integral signaling cascade. Notably, ECT-depleted parasites invaded host cells very poorly (Fig. 3e), suggesting the inability of the mutant to establish a new infection. Since the invasion process is driven by gliding motility, we assayed the latter phenotype (Fig. 3f). Our data disclosed a marked decline in the motile fraction and trail length of the IAA-exposed mutant but not in control samples. The observed defects in the parasite motility, invasion and replication culminated in a highly reduced proliferation of the conditional mutant incubated with auxin. Its tachyzoite count (parasite yield) dropped by ~90% upon IAA treatment compared to the control cultures (Fig. 3g).

### Loss of ECT impairs the inner membrane complex and phospholipid synthesis

**Fig. 4:**
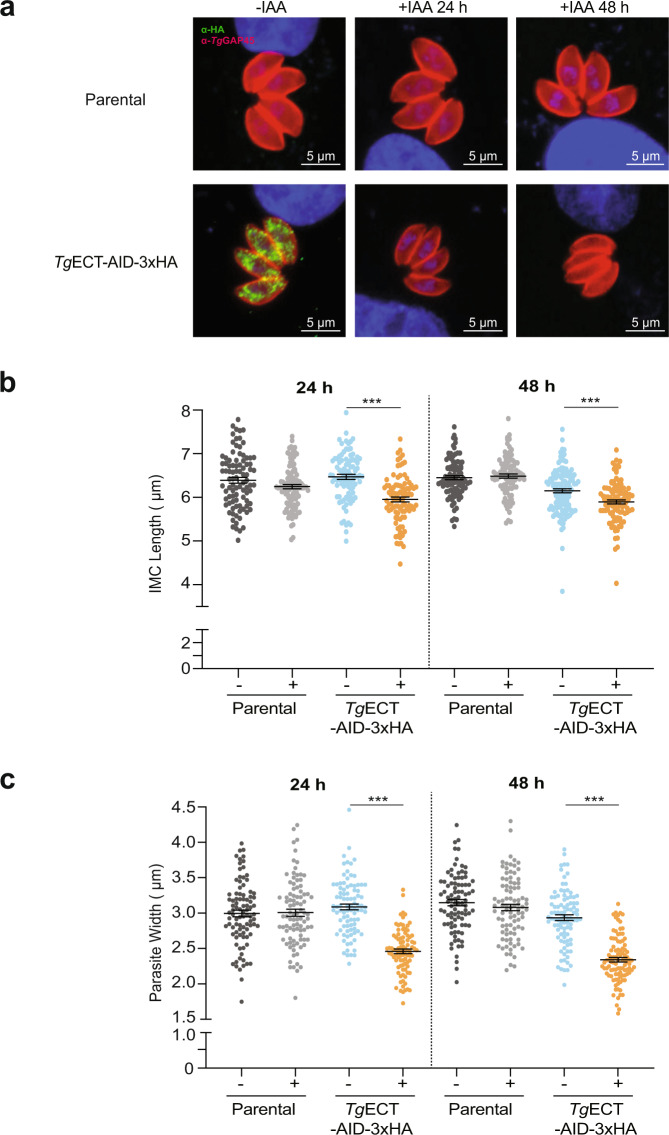
Loss of ECT is associated with anomalies in inner membrane complex. **a** Tachyzoites of the *Tg*ECT-AID-3xHA strain were cultured (−/+ IAA), followed by immunostaining of *Tg*GAP45. Nuclei were visualized by DAPI. **b**, **c** The length of IMC and the width of tachyzoites in the transgenic and parental strains after culturing with or without auxin for 24 h and 48. Images of 30 parasitophorous vacuoles, each with 4 tachyzoites, were analyzed by ImageJ to calculate the indicated parameters (*n* = 3 assays, means ± S.E.; ***p ≤ 0.001).

The apicomplexan motility and locomotion-dependent invasion and egress events are driven by an actin-myosin motor (glideosome) embedded in the inner membrane complex (IMC)^34^. The staining of daughter cells (Fig. 3b) pointed to a malfunctioning organelle, which we examined by visualizing *Tg*GAP45—a well-characterized protein essential for the glideosome function^35,36^—in tachyzoites of the *Tg*ECT-AID-3xHA strain (Fig. 4a). Culturing mutants with IAA for 24 and 48 h resulted in evidently narrow and shortened parasites compared to the parental strain (Fig. 4a). To consolidate our observation, we measured the length and width of *Tg*GAP45-labeled IMC. A significant decrease in these two dimensions was recorded at both time points after IAA treatment. While the length of IMC was shrunk by 10% (Fig. 4b), its width of tachyzoites declined by 25% upon exposure to auxin (Fig. 4c). Presuming a contribution of ECT in membrane biogenesis, we also visualized other organelles by immunostaining, such as micronemes (MIC2), plasma membrane (SAG1), apicoplast (ferredoxin) and mitochondrion (HSP60) (Supplemental Fig. 2). No morphological anomaly was apparent in any of the immunostained organelles. We also deployed transmission electron microscopy to decipher the potential organellar defects in the *Tg*ECT-depleted mutant (Supplemental Fig. 3), which did not show a distinct phenotype compared to untreated control or the parental strain (−/+IAA), ascertaining our initial findings by indirect immunofluorescence assays.

**Fig. 5:**
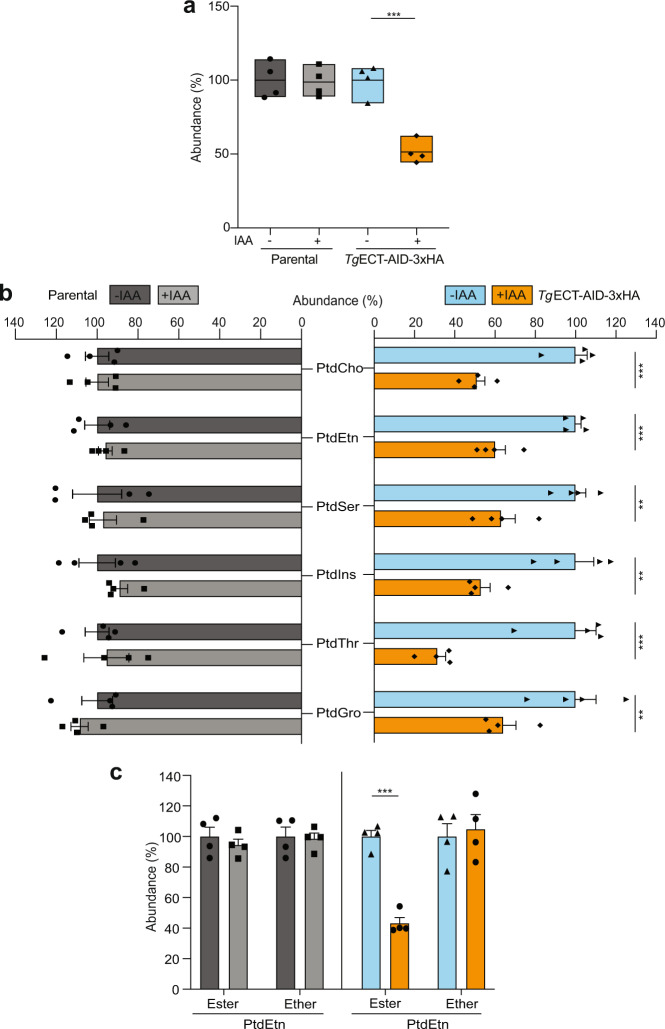
Auxin-induced depletion of TgECT dysregulates major phospholipids in T. gondii. **a** Comparative phospholipid content of the *Tg*ECT-AID-3xHA and parental strains. Parasites were cultured (−/+IAA), followed by lipidomic analysis (*n* = 4 assays). The relative abundance of each phospholipid in IAA-treated samples is presented compared to the untreated control culture (−IAA, 100%). **b** Relative abundance of standard phospholipid classes in tachyzoites of the specified strains (−/+IAA). **c** Changes in ester- and ether-linked PtdEtn pools of the mutant and parental tachyzoites. The data in (**a**–**c**) show the means with S.E. (*n* = 4 assays). Statistical significance was calculated by comparing the +IAA to −IAA samples (**p ≤ 0.01; ***p ≤ 0.001).

Given the putative role of ECT in PtdEtn synthesis, we performed a lipidomic analysis of the *Tg*ECT-AID-3xHA and parental strains (Fig. 5). Total lipids were isolated from parasites, resolved and analyzed by HPLC-MS. Compared to the parental and untreated mutant controls, we witnessed about a 50% decline in the phospholipid content of the ECT-depleted strain (Fig. 5a). A significant decrease in all major phospholipid classes of IAA-treated mutant was seen, whereas no change was apparent in the parental strain (Fig. 5b). The abundance of shown lipids plummeted by 30–50% upon ECT depletion. We also measured levels of the ester and ether-linked PtdEtn (Fig. 5c). None were altered in −/+ IAA cultures of the parental strain. A knockdown of ECT in the mutant caused a decline in ester-PtdEtn but not in ether-lipid (Fig. 5c). Hence, the ratio of ester vs. ether-PtdEtn was changed from 2.6:1 in untreated cultures to 1.1:1 in the ECT-depleted mutant.

### Knockdown of ECT causes differential regulation on ester- and ether-linked lipids

**Fig. 6:**
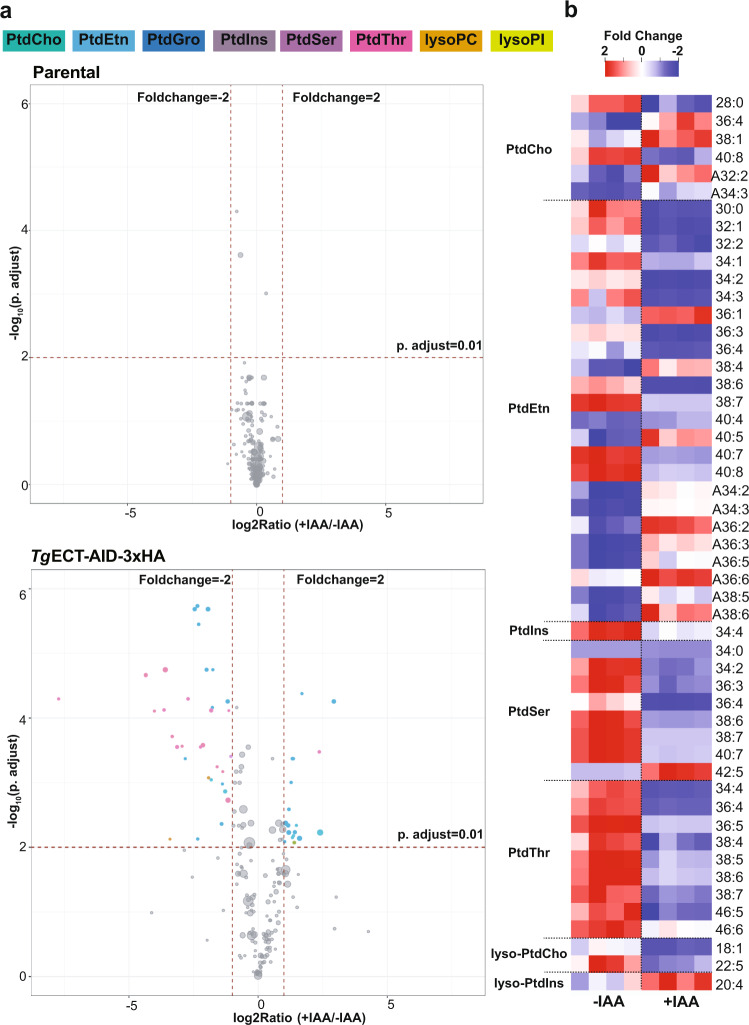
Knockdown of TgECT modulates selected phospholipid species. **a** Volcano plots of the parental and *Tg*ECT-AID-3xHA (*bottom*) strains to depict changes in individual phospholipid species upon IAA exposure. Defined thresholds of fold-change (≥2x) and false-discovery rate-corrected *p* value (≤0.01) were used to identify significantly-altered lipids (*n* = 4 assays). Circles of different sizes, signifying individual species, are scaled to abundance; those above the stated threshold are colored according to phospholipid class, while others are shown in gray. **b** Heatmaps showing changes in lipid species chosen from (**a**). The prefix letter “A” indicates the ether-form of PtdCho and PtdEtn. Lipidomics data are available in the Excel sheets (Supplement Datasheet 1–2).

**Fig. 7:**
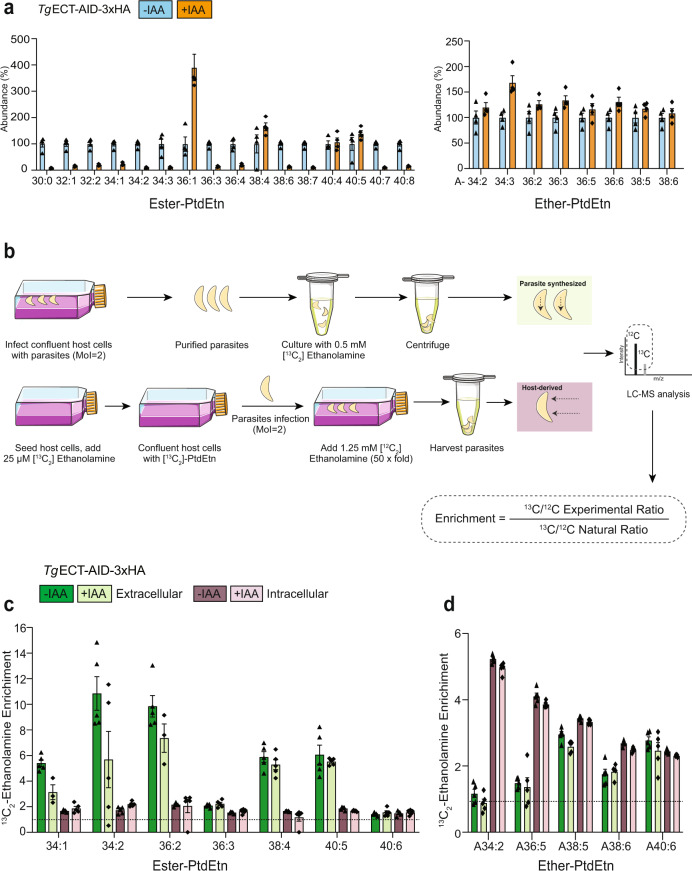
Tachyzoites can synthesize and salvage PtdEtn species. **a** Relative abundance of ester- and ether-linked PtdEtn species significantly altered upon treatment of the *Tg*ECT-AID-3xHA with IAA (Fig. 6). **b** Scheme for the stable isotope labeling of extracellular and intracellular *Tg*ECT-AID-3xHA mutant tachyzoites (−/+IAA) with [^13^C_2_]-ethanolamine and host-derived [^13^C_2_]-PtdEtn, respectively. For the latter assay, HFFs were cultured in [^13^C_2_]-ethanolamine to label PtdEtn, followed by propagation of tachyzoites to test the salvage of [^13^C_2_]-PtdEtn. The incorporation of [^13^C_2_] in ester- and ether-PtdEtn species of purified parasites was judged by lipidomic analysis. **c**, **d** Enrichment of [^13^C_2_]-ethanolamine in ester- and ether-linked PtdEtn species of extracellular and intracellular tachyzoites, as described in (**b**). The data in (**a**, **c**, **d**) show the means with S.E (**a**, *n* = 4 assays; **c**, **d**, *n* = 5 assays).

We next plotted the magnitude of change in all detectable phospholipid species irrespective of abundance as volcano plots to illustrate the fold-change vs. statistical significance upon IAA-induced loss of ECT (Fig. 6a, b). None of the lipid species in the parental strain were affected above a threshold of ≥2-fold (*p* value ≤ 0.01) (Fig. 6a). The *Tg*ECT-AID-3xHA strain, on the other hand, displayed substantial regulation of several lipid species corresponding to PtdCho, PtdEtn, PtdIns, PtdSer, PtdThr, lyso-PtdCho and lyso-PtdIns (Fig. 6b, Supplemental Figs. 4–5). Among downregulated ester-phospholipids, PtdEtn, PtdSer and PtdThr species were most evident (Fig. 6b). A few others, namely C36:1, C38:4, C40:5 PtdEtn, C42:5 PtdSer, C36:4, C38:1 PtdCho and C20:4 lyso-PtdIns were upregulated. Interestingly, ether-lipids belonging singularly to PtdEtn and PtdCho were upregulated or unaltered in the mutant (Figs. 6b, 7a).

The interlinked synthesis of phospholipids motivated us to find patterns in their co-regulation. A loss of ECT altered several species, especially those shared by PtdSer, PtdThr and PtdEtn. C34:2, C38:6, C38:7, C40:7 PtdSer and C38:6, C38:7 PtdThr matched the trend to PtdEtn species (Supplemental Fig. 4), suggesting their synthesis by the base-exchange PSS and PTS enzymes in *T. gondii*^7^. There was barely a common PtdEtn and PtdCho species (Fig. 6b), ratifying the lack of a lipid methyltransferase^5^. Equally, no correlation of PtdEtn species with PtdIns was seen, as expected by sovereign routes of their syntheses^12,25^. Strikingly, some species of PtdCho (36:4, 38:1), PtdEtn (36:1, 38:4, 40:4, 40:5) and PtdSer (42:5) were induced in the ECT-deprived mutant (Supplemental Fig. 3b), pointing to a counterbalancing mechanism. However, none of the specified PtdEtn species was ^13^C_2_-labeled in extracellular tachyzoites or in those grown in pre-labeled host cells (Fig. 7b, c, *see below*). Such ester-PtdEtn species are therefore likely formed by PSDs^10,21^.

### Tachyzoites can salvage ether-linked PtdEtn from the host environment

Unlike its ester form, ether-PtdEtn species were increased or unaffected (Fig. 7a), prompting us to search the enzymes of ether-PtdEtn synthesis in *T. gondii*. As mentioned above, our bioinformatic analysis did not find a suitable candidate in the parasite genome. Therefore, we hypothesized that the Kennedy pathway provides the ester-linked PtdEtn and the production of ether-lipids depends on the parasite milieu. To test this premise, we performed the isotope labeling (^13^C_2_-ethanolamine) followed by lipidomic analysis, which enabled us to distinguish the PtdEtn species synthesized by the parasite from those salvaged from the host cells (Fig. 7b). Akin to previously reported ^13^C_6_-*myo*-inositol labeling of PtdIns^12^, host-free parasites labeled with ^13^C_2_-ethanolamine were analyzed to discern the endogenously-made PtdEtn. At the same time, tachyzoites cultured in pre-labeled host cells were deployed to deduce the occurrence of a salvage pathway.

Lipidomic analysis of the extracellular parasites revealed enrichment of ^13^C_2_-ethanolamine in several ester-PtdEtn species, such as C34:1 (>5-fold), C34:2 (∼11x), C36:2 (∼10x), C38:4 (∼6x) and C40:5 (∼6x) (Fig. 7c), which confirmed the presence of a functional CDP-ethanolamine pathway^5,10^. The IAA-mediated ECT depletion impaired the isotope labeling of C34:1, C34:2, and C36:2 species, consistent with the observed decline in their content (Fig. 7a). By contrast, the ^13^C_2_-labeling of C38:4 and C40:5 PtdEtn were not affected by IAA (Fig. 7c) in accord with their unperturbed or increased levels (Fig. 7a). Tachyzoites grown in ^13^C_2_-ethanolamine-labeled host cells did not show isotopic enrichment in any of the ester-PtdEtn species ruling out salvage of these lipids (Fig. 7c). Inversely, we witnessed an opposite phenomenon for ester-PtdEtn species (A34:2, A36:5) that were ^13^C_2_-enriched (∼4–5x) in intracellularly-grown but not in host-free parasites (Fig. 7d). Other lipid species (A38:5, A38:6, A40:6) were labeled equally under both settings (∼2–3x), and as expected, no change in ^13^C_2_-labeling of ether-PtdEtn was seen upon depletion of ECT. The data entail that ester-PtdEtn species are synthesized primarily de novo using the CDP-ethanolamine pathway, while the parasite salvages ether-linked PtdEtn species from its host cell.

## Discussion

**Fig. 8:**
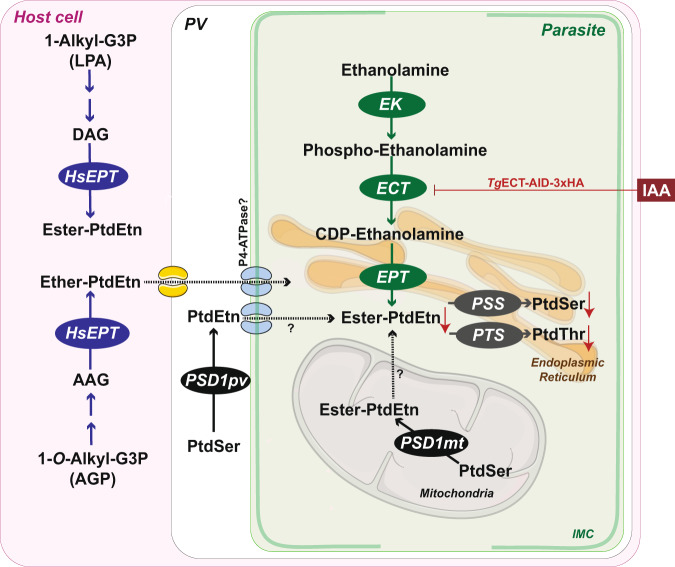
PtdEtn biogenesis in tachyzoites of T. gondii. The model is based on this work and the previous work cited herein. Tachyzoites deploy multiple routes to produce PtdEtn. The demand for ester-PtdEtn is fulfilled primarily *via* the CDP-ethanolamine and PSD1_mt_ pathways. The former drives de novo synthesis of ester-PtdEtn in the ER, whereas the latter generates PtdEtn by decarboxylating PtdSer in the mitochondrion. ECT, located in the parasite cytosol, is the second and rate-limiting enzyme of the CDP-ethanolamine route. It is essential for the parasite survival, and its conditional knockdown in the *Tg*ECT-AID-3xHA mutant by IAA leads to a decline in many PtdEtn, PtdSer and PtdThr species. The latter two lipids are likely made from PtdEtn *via* the base-exchange reactions driven by PSS and PTS enzymes in the ER. Loss of ECT impairs the inner membrane complex, which may underlie defects in gliding motility, invasion and cell division. The parasite’s need for ether-linked PtdEtn is met by salvage from the host cell. Whether intracellular tachyzoites import PSD1_pv_-derived ester-PtdEtn is unclear. P4-ATPases residing on the parasite surface may play a role in the flipping of ether- and ester-linked PtdEtn.

Understanding the metabolic basis of intracellular parasitism in apicomplexan parasites has been central to pathogen-host research. Over the last three decades, *Toxoplasma gondii* has evolved into a model parasite to discover apicomplexan biology due to the relative ease of culture and advanced tools for genome engineering and mutant phenotyping. Albeit actively studied, lipid biosynthesis, trafficking, salvage, sensing and signaling still remain poorly appreciated in Apicomplexa. Exploring a phosphoethanolamine cytidylyltransferase (ECT), this work determined that tachyzoites of *T. gondii* deploy distinct routes to fulfill their need for the ester- and ether-linked PtdEtn species (Fig. 8). While the former lipids are generated through the Kennedy pathway, the second type is salvaged from the host cells. ECT is an essential protein facilitating parasite invasion and replication during the lytic cycle. These features and its phylogenetic divergence from the mammalian homologs make *Tg*ECT an excellent drug target for therapeutic inhibition of acute toxoplasmosis.

Our work suggests the role of the CDP-ethanolamine pathway in making ester-PtdEtn. The levels of many ester-PtdEtn species were reduced in the ECT mutant. The unperturbed and upregulated amounts of other specific lipids indicate the functional presence and contribution of additional routes. Ester-PtdEtn can be generated by PSD1_mt_ and PSD1_pv_ (Fig. 8)^10,21^. Moreover, at least the extracellular tachyzoites are able to salvage PtdEtn^15,20^, and it is plausible that when intracellular, they can acquire PtdSer-derived PtdEtn made in the parasitophorous vacuole by catalysis of PSD1_pv_. Finally, the host endoplasmic reticulum and mitochondria recruited onto the vacuolar membrane are the major sites of PtdSer and PtdEtn synthesis in mammalian cells and possibly serve as an extra source of these lipids. Interestingly, the PSD1_mt_-knockout tachyzoites can survive in prolonged cultures and show upregulation of the CDP-ethanolamine route;^10^ however, the opposite seems not true as ECT is essential for the lytic cycle. We propose that distinct reactions drive the synthesis of discrete ester-PtdEtn species, and those encoded by the Kennedy pathway cannot be equipoised by other means, leading to a lethal phenotype in the ECT mutant.

The physiological relevance of the Kennedy pathway has also been studied in other parasitic protists, including *T. brucei* and *P. bergei*^37,38^. The underlying enzymes are reported to be essential for these parasites even though they harbor functional PSD enzymes. Depletion of *Tb*ECT impaired the parasite proliferation, correlated with a reduced PtdEtn content and unusual morphology in *T. brucei*^37^. These findings mirror the phenotype in the ECT-depleted tachyzoites of *T. gondii*. Conditional loss of *Tg*ECT abrogated the parasite invasion and replication, concurrent with abnormal IMC that can be attributed to dysregulated phospholipid composition (possibly PtdEtn species). Hosting glideosome, a motor complex driving the parasite motility^34^, IMC is crucial for the lytic cycle. Its perturbation upon loss of *Tg*ECT may result in dysfunctional glideosome, leading to impaired motility, invasion and egress in tachyzoites. Besides, a skewed ratio of ester to ether-linked PtdEtn in the mutant may affect the membrane fluidity and cytokinesis, as described in mammalian cells^23^.

Our data also suggest that PtdEtn serves as a donor for synthesizing PtdSer and PtdThr *via* ethanolamine-to-serine or threonine exchange reaction catalyzed by PSS and PTS proteins, respectively (Fig. 8). Notably, a few PtdEtn species (36:1, 38:4, 40:4, 40:5) were unaffected or even elevated upon depletion of ECT. They are likely made by PtdSer decarboxylation, which merits lipidomic analysis of the PSD1_mt_ and PSD1_pv_ mutants. No parallels were evident in the modulation of PtdCho and PtdIns species with that of PtdEtn, reverberating with the independent routes of syntheses for the former two phospholipids^4,5,12^. Importantly, this study reveals the salvage of host-derived ether-PtdEtn species by intracellular parasites (Fig. 8). While tachyzoites are known to produce diacylglycerol for making ester-PtdEtn^18^, a bona fide alkyl-glycerone phosphate synthase for producing the alkyl-glycerol scaffold of ether-PtdEtn could not be found in the parasite genome. The presence of ether-linked lipids in tachyzoites is enigmatic. They are reported to have “redox” effects in mammalian cells^22,24^. Certain cultured cells lacking them become more sensitized to oxidative damage. Equally, the pro-oxidant feature of these lipids has also been described^39^. Future work should focus on dissecting the salvage machinery and functional importance of ether-lipids in tachyzoites of *T. gondii*.

In conclusion, this work demonstrates *Tg*ECT as a vital enzyme contributing to the synthesis of ester-PtdEtn. Its conditional depletion impairs the membrane biogenesis, replication and invasion in tachyzoites. We reveal a collaboration of endogenous synthesis, interconversion and salvage pathways to produce PtdEtn. In particular, the data show previously-unknown scavenging of host-derived ether-linked PtdEtn species by the parasite and offer a sound basis for therapeutic targeting of PtdEtn synthesis in *T. gondii*.

## Methods

### Biological reagents and resources

The RH*∆ku80∆hxgprt-*TIR1 strain of *T. gondii*, the *pLinker-AID-3xHA-HXGPRT* plasmid for AID-3xHA tagging^40^, anti-*Tg*ALD and anti-*Tg*MIC2 antibody were provided by David Sibley (Washington University, St. Louis, USA). Other primary antibodies binding to *Tg*GAP45, *Tg*HSP90, *Tg*IMC3, and *Tg*Ferredoxin were offered by Dominique Soldati-Favre (University of Geneva, Switzerland), Sergio O. Angel (IIB-INTECH, Argentina), Marc-Jan Gubbels (Boston College, USA) and Frank Seeber (Robert-Koch Institute, Berlin), respectively. Primary antibody against *Tg*HSP60 and the pig anti-*Tg* serum were produced in the State Key Laboratory of Agricultural Microbiology (Wuhan, China). Other primary antibodies against the hemagglutinin (HA) epitope and *Tg*SAG1 were obtained from Sigma-Aldrich (Germany) and ThermoFisher Scientific (Germany), respectively. The secondary antibodies for immunostaining (Alexa488, Alexa594, IRDye 680RD, 800CW) and oligonucleotides (Supplemental Table 1) were purchased from Life Technologies (Germany). The cell culture reagents were procured from PAN Biotech (Germany). Other standard chemicals were supplied by Sigma-Aldrich and Carl Roth (Germany). The reagent kits for cloning were acquired from Analytik Jena and Life Technologies (Germany). Lipid standards were delivered by Avanti Polar Lipids (USA).

### Parasite and host cell cultivation

Human foreskin fibroblasts (HFFs) were used as the host cells to propagate tachyzoites of *T. gondii*. Cells were harvested by trypsinization (0.25% trypsin-EDTA) and seeded in flasks, dishes or coverslips, as per the requirement of individual assays. The confluent monolayers were infected on alternate days to maintain tachyzoites. Cultures were performed in DMEM (Dulbecco’s Modified Eagle’s Medium) supplemented with glucose (4.5 g/L), fetal bovine serum (10%, PAN Biotech, Germany), glutamine (2 mM), sodium pyruvate (1 mM), penicillin (100 U/mL), streptomycin (100 μg/mL) and minimum Eagle’s non-essential amino acids (100 μM each) at 37 °C and 5% CO_2_ in a humidified incubator. For most assays, tachyzoites were released from late-stage parasitized cultures by scraping and squirting through 23- and 27-gauge syringes. The extracellular parasites were utilized directly for downstream assays.

### Construction of transgenic lines

Tachyzoites (~10^7^) were transfected with 10 µg plasmid constructs in filter-sterile cytomix (120 mM KCl, 0.15 mM CaCl_2_, 10 mM K_2_HPO4/KH_2_PO4, 25 mM HEPES, 2 mM EGTA, 5 mM MgCl_2_ supplemented with fresh 5 mM glutathione and 5 mM ATP; pH 7.6). Parasites were then selected with the drug corresponding to the selection marker encoded by the transfected plasmid. The drug-resistant transgenic parasites were cloned by limiting dilution in 96-well plates containing HFF cells. Individual clones with desired gene manipulation were identified by screening PCR and immunostaining assays. To generate the *Tg*ECT-AID-3xHA strain, a donor amplicon comprising the AID-3xHA-*Tg*GRA1-3′UTR and DHFR-TS selection marker flanked by short (40 bp) homologous arms for a crossover at the ECT locus was co-transfected with a CRISPR-Cas9 construct in the RH*∆ku80∆hxgprt-*TIR1 strain. To generate the *Tg*ECT-smHA strain, a plasmid construct expressing Cas9 and *Tg*ECT-specific *sg*RNA was transfected with a PCR amplicon comprising 10xHA and DHFR-TS expression cassette in the RHΔ*ku80*-*hxgprt*^*−*^ strain. The mutant parasites were selected by 1 µM pyrimethamine and PCR-screened for the AID-3xHA or smHA tagging of ECT.

### Indirect immunofluorescence assays

The assay was executed, as reported earlier^41^. HFF cells were seeded and grown to confluence on the glass coverslips placed in 24-well plates. Freshly-released parasites were used to infect HFF monolayers. Parasitized host cells were washed with PBS and fixed in 4% paraformaldehyde for 15 min, followed by neutralization in 0.1 M glycine/PBS. Samples were permeabilized by 0.2% Triton-X100/PBS (20 min) and blocked with 2% BSA prepared in 0.2% Triton-X100/PBS (20 min). Cells were finally stained with the matching primary (α-HA, 1:3000; α-*Tg*GAP45, 1:5000; α-*Tg*IMC3, 1:2000; α-*Tg*ALD, 1:1000; α-*Tg*Ferredoxin, 1:500; *Tg*SAG1, 1:1000; anti-*Tg* porcine serum, 1:1000, *Tg*MIC2, 1:1000) and secondary (Alexa488, Alexa594, 1:3000) antibodies for 1 h each. Samples were washed by PBS between different treatment steps and mounted in DAPI-Fluoromount G for imaging (Carl-Zeiss, Germany).

### Lytic cycle assays

Phenotypic assays were set up with fresh syringe-released parasites, as reported previously^7,12,32^. Briefly, plaque assays were performed in 6-well plates by infecting confluent HFF monolayers with 200 parasites/well, followed by unperturbed incubation (−/+ 100 µM IAA in 0.1% ethanol) for 7 days. The carrier solvent was included in untreated samples. Cultures were fixed with cold methanol (15 min) and stained with crystal violet (15 min). Plaques were quantified using the ImageJ suite (NIH, Bethesda). For replication and endodyogeny assays, the confluent HFF monolayers on coverslips were infected (MoI:1, 24 h, 40 h), followed by fixation and staining with α-*Tg*IMC3 and α-*Tg*GAP45 antibodies. The cell division was assessed by enumerating parasites replicating within their parasitophorous vacuoles. To determine the gliding motility, tachyzoites were pre-cultured with IAA for 48 h (if applicable). Afterward, 4 × 10^5^ parasites suspended in Ca^2+^-free Hank’s balanced salt solution (−/+ IAA) were settled (400 *g*, 5 min, room temperature), followed by incubation (15 min, 37 °C) on coverslips coated with 0.01% BSA. To visualize the parasites and trails, samples were stained by α-*Tg*SAG1 and Alexa488 antibodies. Motile fractions were estimated directly on the microscope, while trail lengths were quantified using the ImageJ software.

For the invasion test, tachyzoites were pre-cultured in 100 µM IAA or the carrier solvent for 40 h and then used to infect the host cells on coverslips (MoI:10, 1 h). Samples were fixed, neutralized and stained, as reported elsewhere^32^. The non-invaded (extracellular) parasites were immunostained for *Tg*SAG1 before detergent permeabilization of cultures. Samples were washed by PBS, permeabilized and stained for *Tg*GAP45 protein to visualize the invaded (intracellular) tachyzoites. Cultures were washed with PBS, labeled with secondary antibodies (Alexa488, Alexa594), and mounted in DAPI-Fluoromount G for fluorescent imaging (Carl-Zeiss, Germany). The percentage of invaded parasites (invasion efficiency) was counted by dual-colored parasites. For natural egress assays, HFFs cultured on coverslips were infected with parasites (MoI:2, 40 h/64 h, −/+ 100 µM IAA), followed by two-step staining (as in invasion assay) to distinguish the disrupted and intact vacuoles^32^.

A single natural egress experiment required us to check the parasite vacuole at two time points, one before egress to determine the total PVs (40 h) and one after egress to score the remaining PVs (64 h). At 40 h, tachyzoites were about to but not egressed, whereas at 64 h, all parasites of the parental strains were egressed. The egress rate (%) was calculated as [number of intact PVs (40 h) – number of intact PVs (64 h)]/number of total PVs (40 h) × 100. For induced egress, the parasitized cultures (MoI:1, 24 h) were treated with 500 µM zaprinast (30 min). Here, an earlier time point (24 h infection, 8-16 parasites/vacuole) was chosen to ensure that no egress occurred under uninduced condition. To quantify the parasite yield, host cells (10^6^) in culture dishes (3 cm) were infected by tachyzoites (10^6^ parasites, MoI: 1) and progeny were counted after syringe-release (27 G) at 48 h.

### Lipid extraction and lipidomic analysis

We selected 48 h infection for the sample collection based on the parasite yield assay. Lipids were extracted from fresh pellets of purified tachyzoites (1 × 10^7^/sample), as reported earlier^7^. Isolated lipids were dried under a nitrogen stream and dissolved in 100 μL of chloroform and methanol (1:1), 10 μL of which was injected onto a Acquity BEH C18 UPLC column (2.1 × 100 mm, 1.7 µm) maintained at 60 °C. Separation of lipid species was achieved with a gradient of methanol and acetonitrile in water, both containing 2.5 mM ammonium acetate. The gradient elution, at a flow rate of 600 µL/min, was programmed as follows (time in min, % B): (0, 12.5), (7.5, 100), (14, 100), (14.1, 87.5), (17, 87.5). The effluent was subjected to heated electrospray ionization in a Sciex X500R QToF instrument. Data-dependent MS2 spectra were collected (MS1 range 400-1050 amu; precursor >600 amu; MS2 range 50-1050 amu) with an accumulation time of 150 ms, collision energy of 35 V and a CE spread of 15 V. Data processing was done using the *Analytics* module of SciexOS and MSDial^42^.

### Stable isotope labeling

The assay was performed as described previously for [^13^C_6_]-*myo*-inositol^12^. To label extracellular parasites with [^13^C_2_]-ethanolamine (Sigma-Aldrich, Germany), the *Tg*ECT-AID-3xHA mutant was precultured without or with 100 μM IAA for 48 h, followed by syringe-release, PBS washing and filtering through 5 μm filter to remove the debris. Parasites (1 × 10^7^) were then suspended in 100 μL DMEM (−/+ IAA) containing 0.5 mM [^13^C_2_]-ethanolamine and incubated at 37 °C for 6 h before lipidomic analysis. For intracellular labeling of parasites in HFFs harboring [^13^C_2_]-PtdEtn, host cells in T-175 flasks were grown to confluence in 25 μM [^13^C_2_]-ethanolamine. Cultures were washed by PBS to remove the excess isotope from media and infected with parasites in the presence of 50× excess (1.25 mM) of ethanolamine to preclude the inclusion of intracellularly accumulated isotope into endogenously-produced PtdEtn of the proliferating tachyzoites. The progeny parasites were analyzed for the ester- and ether-linked PtdEtn species.

### Transmission electron microscopy

The HFF monolayers in T75 flasks were infected with tachyzoites (MoI = 2) for 48 h. Parasitized cells were scarped, washed with PBS and fixed overnight in 2.5% (v/v) glutaraldehyde/PBS (0.1 M, pH 7.2) at 4 °C. Samples were washed with PBS (3×, 30 min at room temperature), post‐fixed in 1% (w/v) osmium tetroxide for 2 h, rewashed with PBS for 30 min, and dehydrated in a graded series of acetone concentrations. Dehydrated blocks were progressively infiltrated and embedded in Spurr resin (SPI Chem, USA), and then polymerized at 65 °C for 48 h. Samples were sliced into ultrathin sections (60–70 nm thick) with a diamond knife, stained with 2% uranyl acetate, and imaged at 80 kV using a transmission electron microscope (H‐7650, Hitachi, Japan).

### Statistics and reproducibility

Unless specified otherwise, data shown in graphs are presented as the mean with S.E. from three experiments using representative parasite clones. Statistical analyses were performed using the GraphPad Prism program (CA, USA). Significance was tested by unpaired two-tailed *t*-test with equal variance (*p ≤ 0.05, **p ≤ 0.01, ***p ≤ 0.001). We introduced the false-discovery rate for the multiple comparison testing.

### Reporting summary

Further information on research design is available in the Nature Portfolio Reporting Summary linked to this article.

## Supplementary information

Supplemental Information

Description of Additional Supplemental Files

Supplemental Data 1

Supplemental Data 2

Reporting Summary

## Data Availability

The data generated and/or analyzed during this study are provided in the main paper (Figs. 1–8) and supplementary files (Supplemental Figs. 1–6, Table 1). The original gel, blot and plaque images are shown in the Supplemental Fig. 6. The source data for the graphs and images can be found in the Supplemental Excel files (Supplemental Data 1–2). All resources are also available from the authors upon reasonable request.
