# Supplementary material for: Synthesis vs. salvage of ester- and ether-linked phosphatidylethanolamine in the intracellular protozoan pathogen Toxoplasma gondii
Source: Commun Biol. 2023 Mar 22;6:306. doi: 10.1038/s42003-023-04664-x (PMC10033509; doi:10.1038/s42003-023-04664-x)
Supplement: Supplementary file 1 — Supplemental Information [file 42003_2023_4664_MOESM1_ESM.pdf]

Supplemental Figure 1

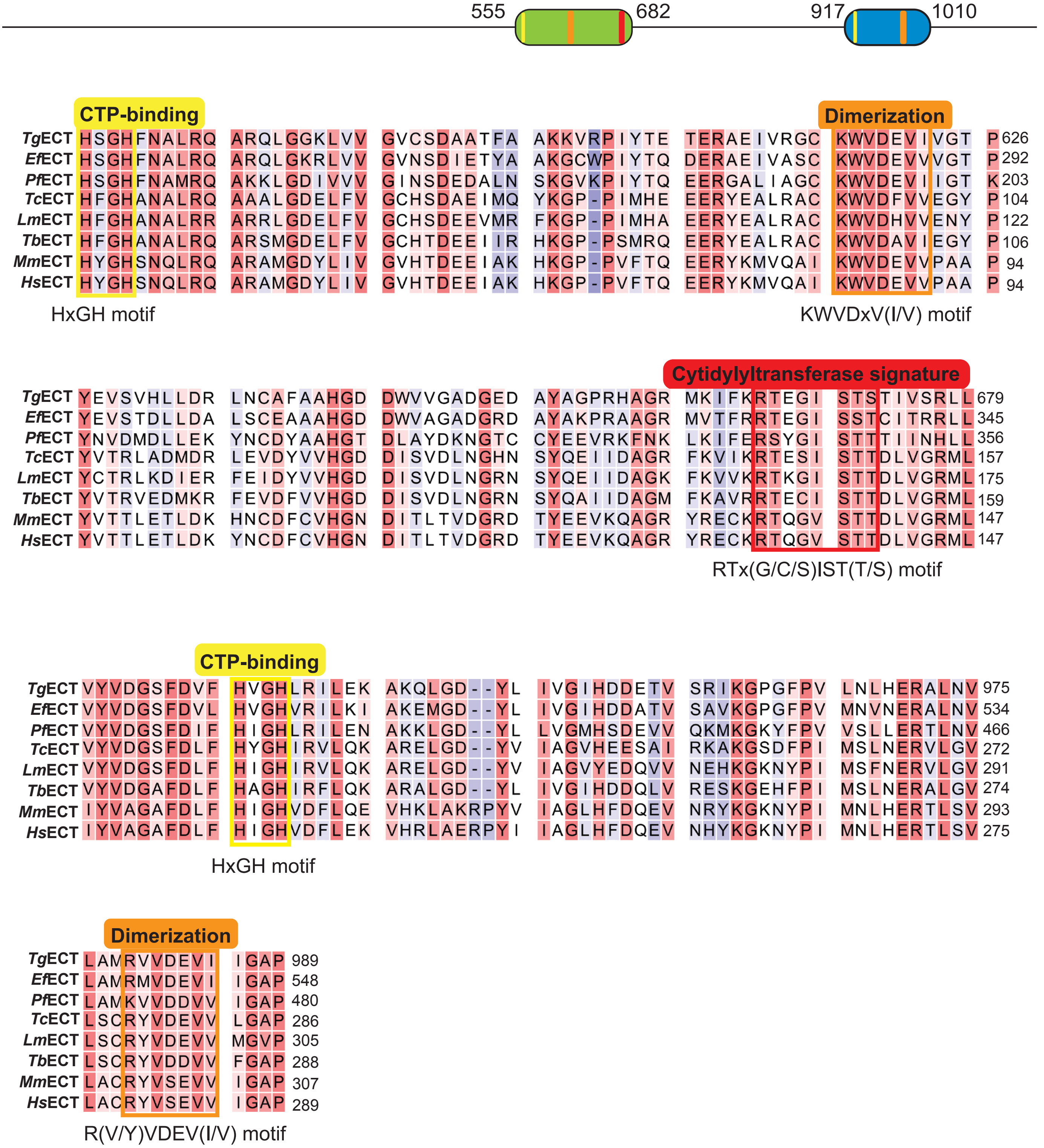

Figure S1: Multiple sequence alignment of *Tg*ECT with homologs from selected organisms. The primary structure of *Tg*ECT shows the position of predicted cytidylyltransferase domains and signature motifs. The upper and lower alignment correspond to the N- and C-terminal CT domains, which fold together into a functional protein. IDs: *Toxoplasma gondii*, *Tg*ECT (TGGT1_310280); *Eimeria falciformis*, *Ef*ECT (EfaB_PLUS_7742.g777); *Plasmodium falciparum*, *Pf*ECT (Pf3D7_1347700); *Trypanosoma brucei*, *Tb*ECT (Tb927.11.14140); *Trypanosoma cruzi*, *Tc*ECT (TcCLB.511727.120); *Leishmania mexicana*, *Lm*ECT (LmjF.32.0890); *Homo sapiens*, *Hs*ECT (Q99447); *Mus musculus*, *Mm*ECT (Q922E4).

Supplemental Figure 2

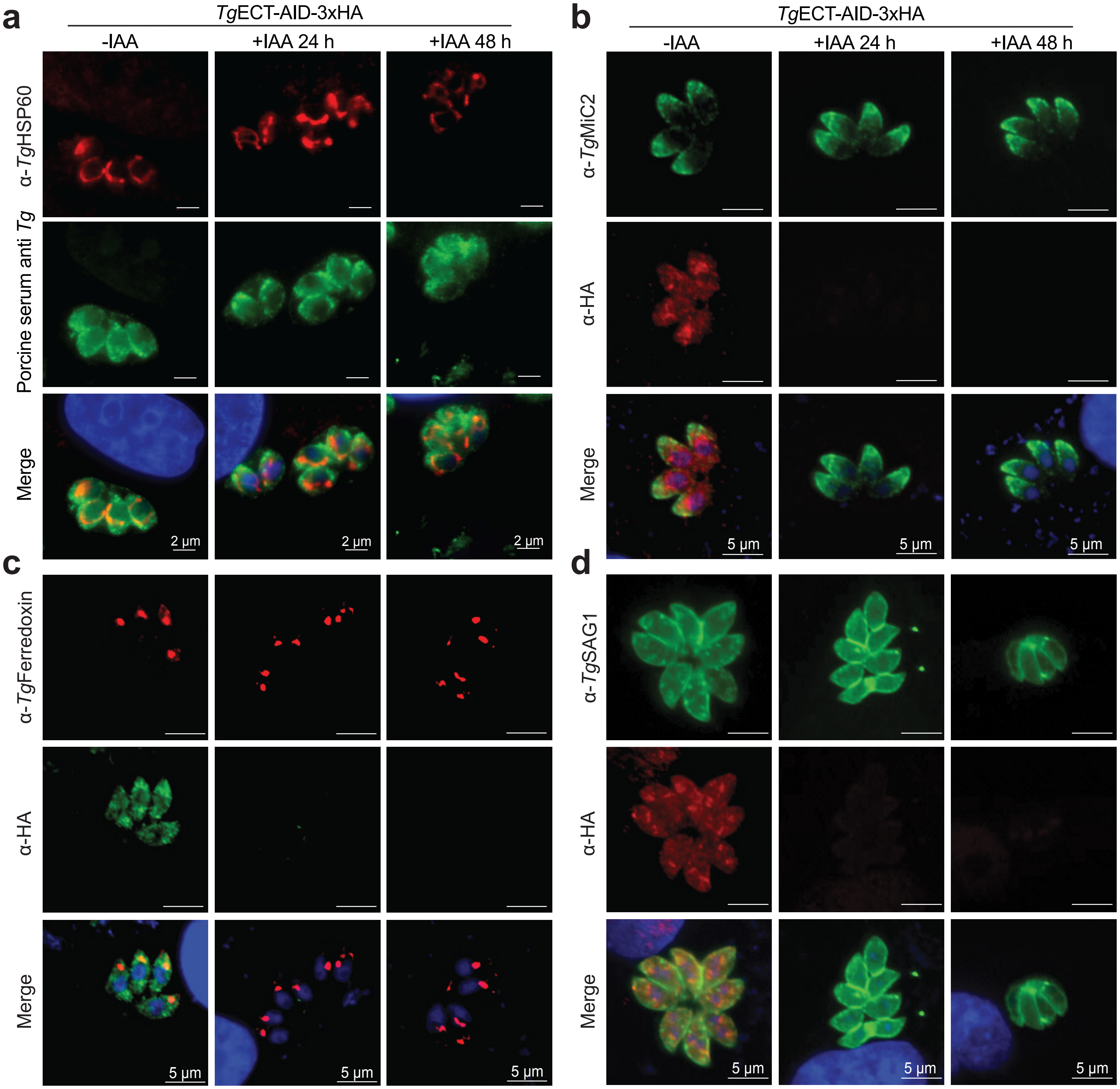

Figure S2: Immunostaining of selected organelles in the *Tg*ECT-AID-3xHA mutant. The antibodies recognizing the parasite mitochondrion (α-*Tg*Hsp60, a), micronemes (α-*Tg*Mic2, b), apicoplast (α-*Tg*Ferredoxin, c) and plasma membrane (α-*Tg*Sag1, d) were used to stain intracellular tachyzoites along with the porcine anti-*Tg* serum and α-HA, as indicated. Parasites were cultured without (-IAA) or with IAA (+IAA for 24 h and 48 h).

Supplemental Figure 3

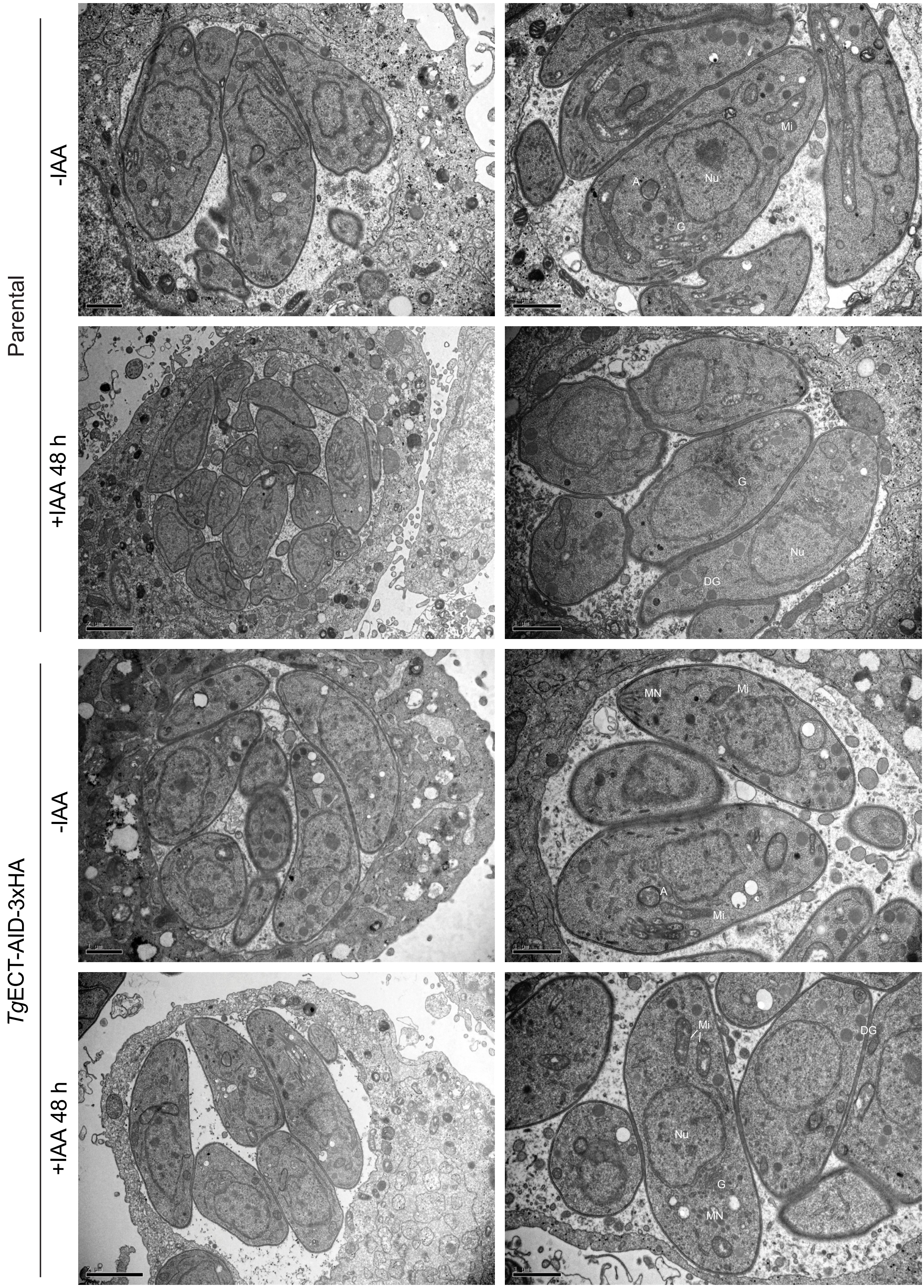

Figure S3: Transmission electron microscopy of the parental and *Tg*ECT-AID-3xHA strains. Nu, nucleus; Mi, mitochondria; A, apicoplast; G, Golgi network; DG, dense granule; MN, microneme.

Supplemental Figure 4

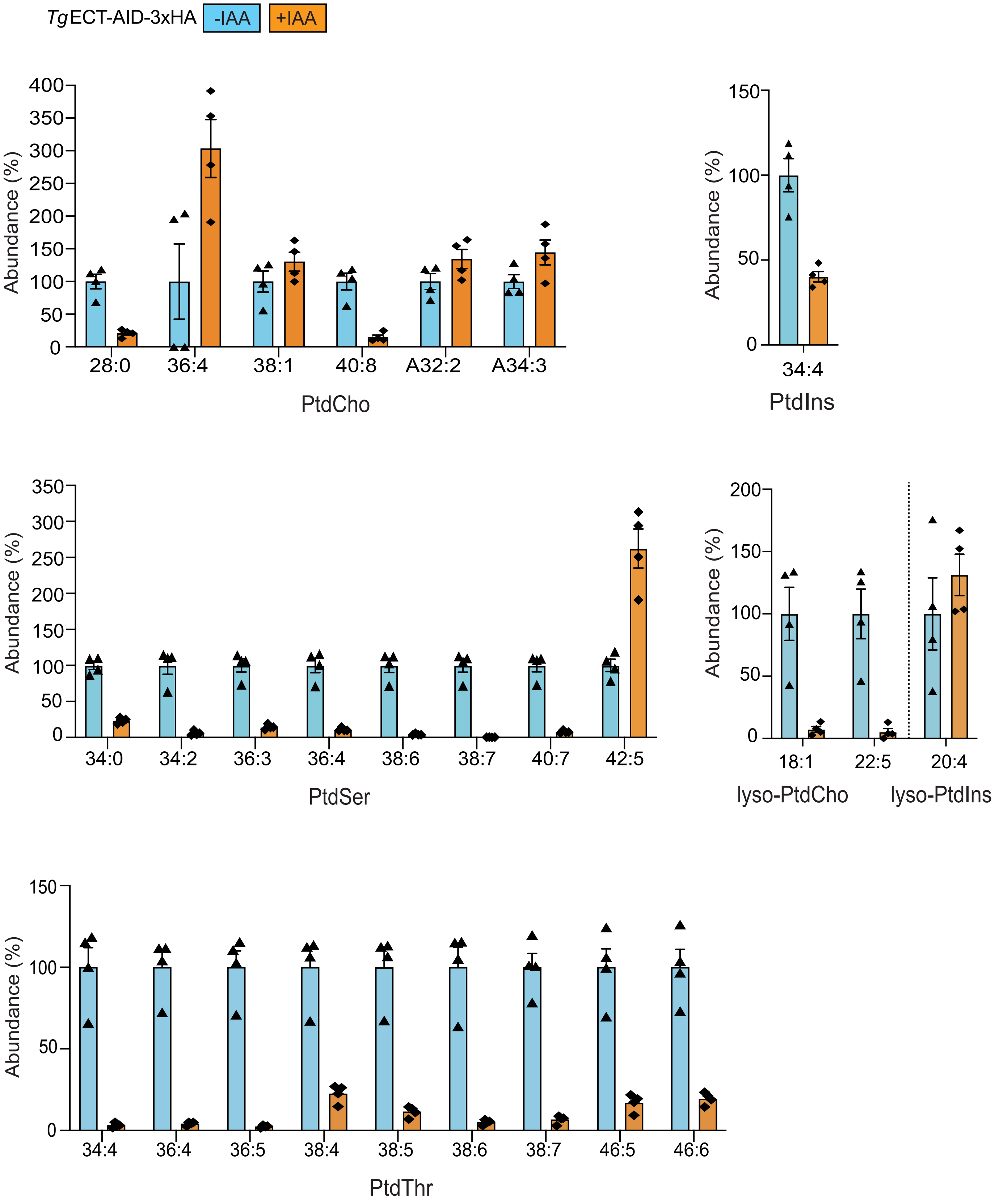

Figure S4: Phospholipids dysregulated in tachyzoites of the *Tg*ECT-AID-3xHA mutant. Lipid species (-/+IAA) depicted here are based on the data presented in Figure 6.The data in bar graphs show the means with S.E. (n =4 assays).

Supplemental Figure 5

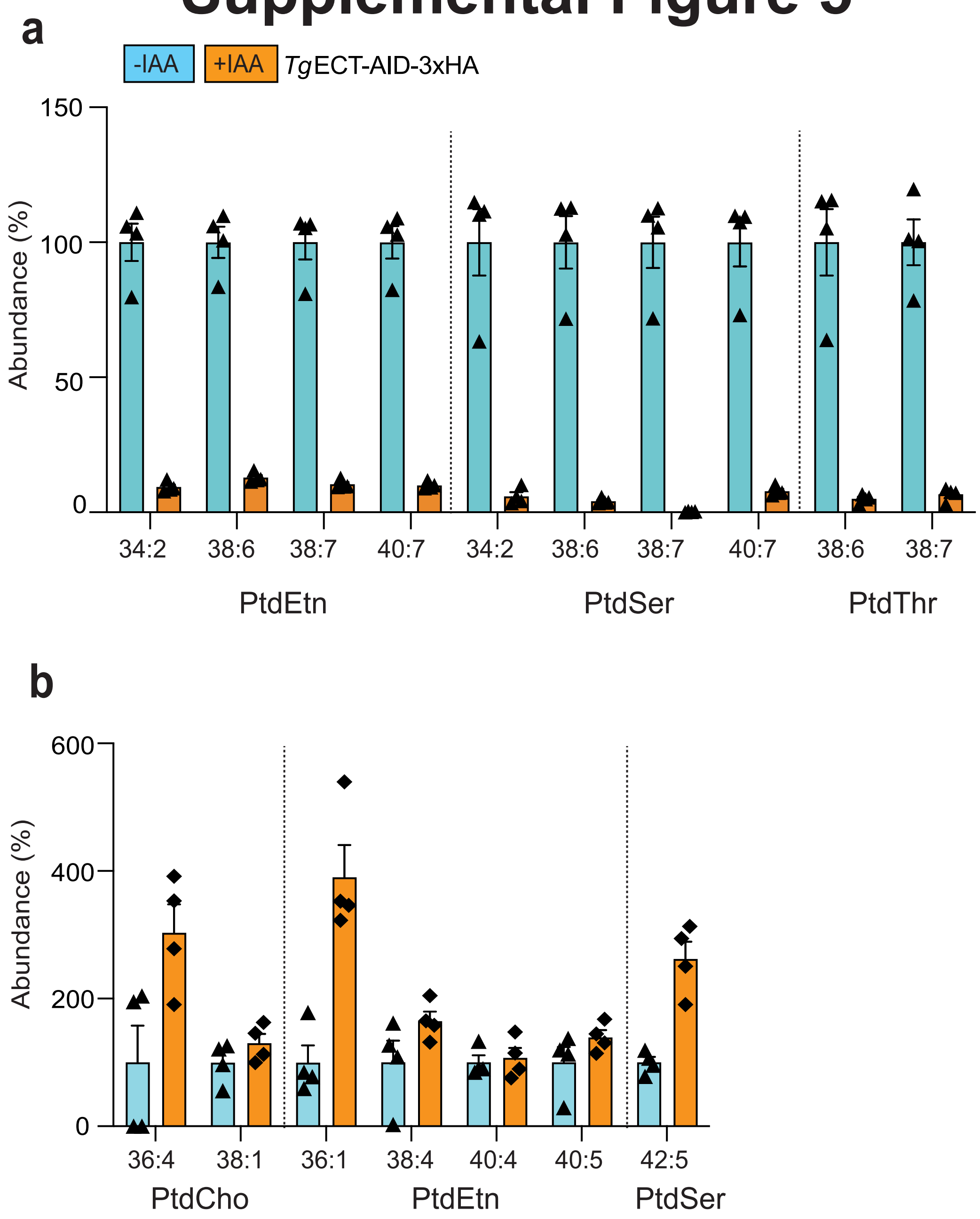

Figure S5: Selected phospholipid species upon depletion of ECT in tachyzoites. The bar graphs show lipid species significantly decreased (a) or increased (b) upon depletion of ECT in the presence of IAA. For additional details, refer to Figure 6. The data in bar graphs show the means with S.E. (n =4 assays).

Supplemental Figure 6

Figure 1d

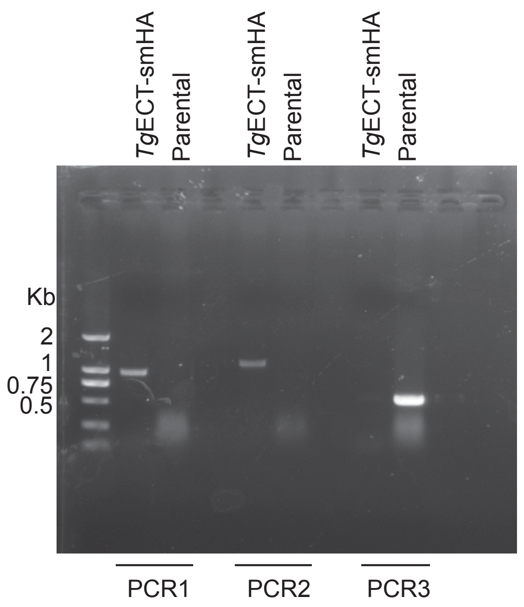

Figure 1e

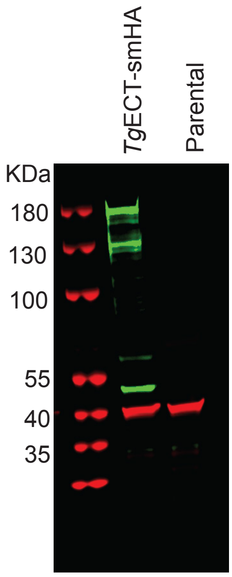

Figure 2b

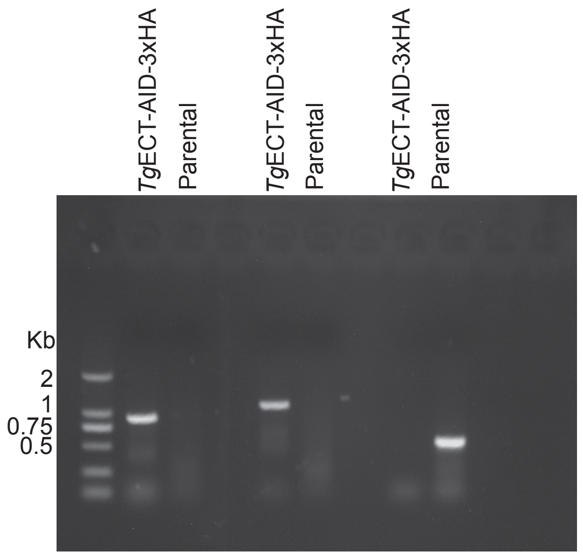

Figure 2d

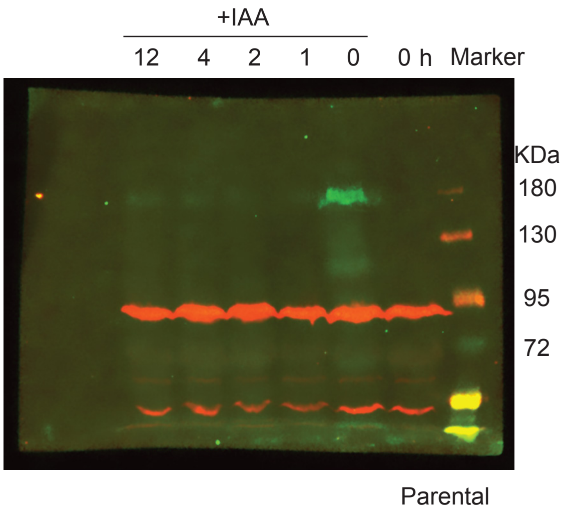

Figure 2f

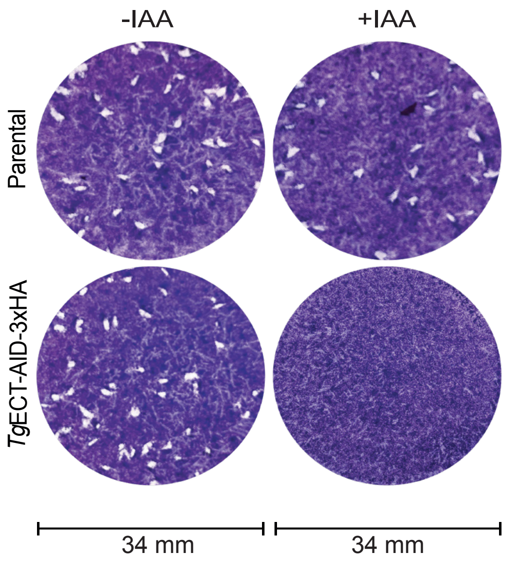

Figure 3a

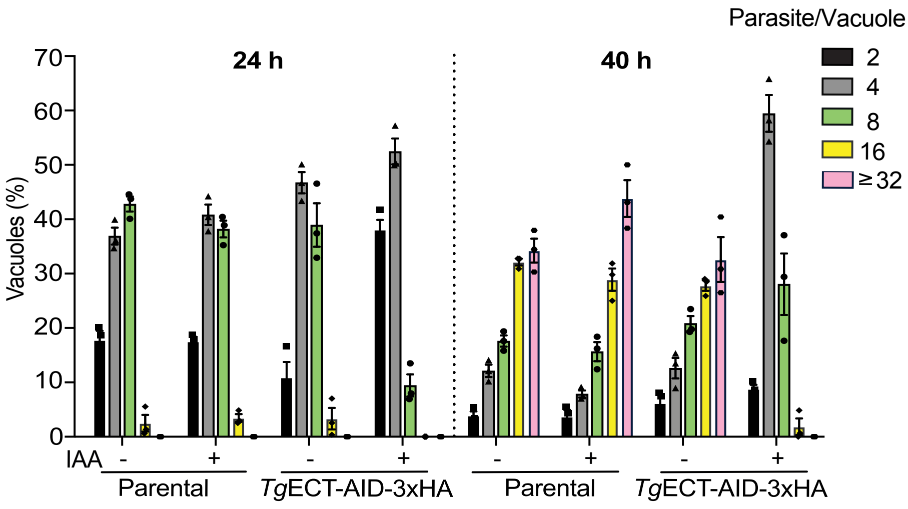

Supplemental Table 1

Primer	Sequence	Purpose
*Tg*ECT-AID-DHFRTS-F	CGAACAAGAGCAGGGACAGATGGTGTCTCTGACAGAACTCATGGGCAG TGTCGAGCTG	Amplifying donor fragment for CRISPR-Based homology direct repair
*Tg*ECT-AID-DHFRTS-R	AAACGGAGAGAACCAAAACTTGACAAAGTCGCGTATCACGTGTCACTG TAGCCTGCCAGA	
*Tg*ECT-smHA-F	CGAACAAGAGCAGGGACAGATGGTGTCTCTGCAGAACTCGGCTCGACG AGGATGTACCC	Amplifying donor fragment for *Tg*ECT-smHA construction
*Tg*ECT-smHA-R	AAACGGAGAGAACCAAAACTTGACAAAGTCGCGTATCACGGACCATGAT TACGCCAAGCG	
pU6-TgECT-HA-AID-F	AAGTTGAATGGATTGATGGACGCGGG	Preparing gRNAs required for CRISPR-Cas9 recognition
pU6-TgECT-HA-AID-R	AAAACCCGCGTCCATCAATCCATTCA	
smHA-ECT-PCR1-F<br>AID-ECT-PCR4-F	GTGTTGCTGTCTTTGCATGCAG	Screening PCR for 5’ homologous recombination
smHA-ECT-PCR1-R	CTTTCGAAAGCACGCTCTGG	
AID-ECT-PCR4-R	AACTTTTCTACATATGCCCGGG	
smHA-ECT-PCR2-R<br>AID-ECT-PCR5-R	GTTGGCGCCTCAGCTCGCTA	Screening PCR for 3’ homologous recombination
smHA-ECT-PCR2-F	GCTGGACTGTTGCTGTCTGC	
AID-ECT-PCR5-F	CTACGTCCCGCACGGACGAA	

Table S1: Oligonucleotides used in this study
