# Supplementary material for: Synthesis vs. salvage of ester- and ether-linked phosphatidylethanolamine in the intracellular protozoan pathogen Toxoplasma gondii
Source: Commun Biol. 2023 Mar 22;6:306. doi: 10.1038/s42003-023-04664-x (PMC10033509; doi:10.1038/s42003-023-04664-x)
Supplement: Supplementary file 2 — Description of Additional Supplemental Files [file 42003_2023_4664_MOESM2_ESM.pdf]

Description of Additional Supplemental Files

File name: Supplemental Data 1
Description: The source data behind the graphs in the paper.

File name: Supplemental Data 2
Description: The source data underlying Fig 6a.
